# Towards an understanding of the structure and function of MTA1

**DOI:** 10.1007/s10555-014-9513-5

**Published:** 2014-10-29

**Authors:** Christopher J. Millard, Louise Fairall, John W. R. Schwabe

**Affiliations:** Henry Wellcome Laboratories of Structural Biology, Department of Biochemistry, University of Leicester, Leicester, LE1 9HN UK

**Keywords:** Metastasis associated protein 1, Corepressor complexes, Chromatin, Inositol phosphate, Transcriptional regulation

## Abstract

Gene expression is controlled through the recruitment of large coregulator complexes to specific gene loci to regulate chromatin structure by modifying epigenetic marks on DNA and histones. Metastasis-associated protein 1 (MTA1) is an essential component of the nucleosome remodelling and deacetylase (NuRD) complex that acts as a scaffold protein to assemble enzymatic activity and nucleosome targeting proteins. MTA1 consists of four characterised domains, a number of interaction motifs, and regions that are predicted to be intrinsically disordered. The ELM2-SANT domain is one of the best-characterised regions of MTA1, which recruits histone deacetylase 1 (HDAC1) and activates the enzyme in the presence of inositol phosphate. MTA1 is highly upregulated in several types of aggressive tumours and is therefore a possible target for cancer therapy. In this review, we summarise the structure and function of the four domains of MTA1 and discuss the possible functions of less well-characterised regions of the protein.

## Introduction

The class I histone deacetylase (HDAC) corepressor complexes are multi-protein assemblies that contain one or more enzymes that modify chromatin, components with nucleosome or DNA binding activity and proteins that function to provide a scaffold to the complex. Five major HDAC-containing corepressor complexes have been described and include nucleosome remodelling and deacetylase (NuRD) [1, 2], COREST [3], Sin3A [4, 5], MIDAC [6] and NCoR/SMRT [4, 7]. Understanding the distinct features and architectures of these complexes is likely to be important for the design of specifically targeted therapeutics.

Metastasis-associated protein 1 (MTA1) and its homologues MTA2 and MTA3 are essential components of the NuRD corepressor complex (reviewed in [8–10]). The MTA proteins do not have any intrinsic enzymatic activity but play a key role in stabilising and assembling the complex. MTA1 was first isolated from a metastatic breast cancer cell line and has since been shown to be upregulated in several other metastatic human cancers [11–13]. Elevated expression levels of MTA1 are strongly associated with the growth of aggressive endometrial, breast and ovarian cancers, and can be used as a prognosis marker in the progression of human cancers [14–16].

**Fig. 1:**
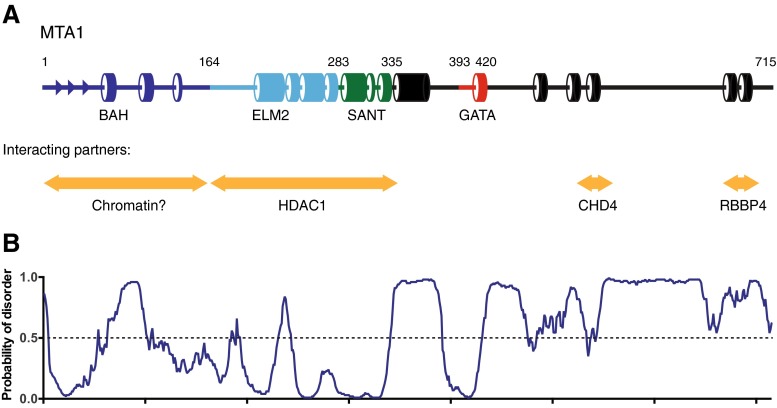
The domain structure of MTA1. **a** Illustration of the secondary structure of MTA1 highlighting the α-helices and β-strands as predicted by PSIPRED [65]. *Orange arrows* indicate the regions of MTA1 that interact with other binding partners. **b** Disorder prediction for MTA1. The four characterised domains fall into ordered regions whereas much of the rest of the protein is predicted to be intrinsically disordered (calculated using DISOPRED3 [66]) (colour figure online)

Over half of the residues of MTA1 are predicted to be intrinsically disordered, and this has presented a major challenge in the structural characterisation of this protein (Fig. 1). However, significant progress has been made in studying domains of MTA1 in complex with other proteins from the NuRD complex. In this review, we examine the structural insights that have been gained for MTA1, MTA2 and MTA3. We also discuss domains and motifs of MTA1 that have not been structurally characterised, but for which structural information is available from related proteins.

## The NuRD corepressor complex

The NuRD corepressor complex consists of six protein subunits: HDAC1/2, MTA1/2/3, p66 α/β, histone-binding protein  4/7 (RBBP4/7), methyl-CpG-binding protein 2/3 (MBD2/3) and chromatin-helicase-DNA-binding protein 3/4 (CHD3/4). Deacetylase and helicase activities are combined in this complex. HDAC1 and HDAC2 are single domain deacetylase enzymes that remove the acetyl group from modified lysines resulting in an extra positive charge on histone tails. This leads to a more compact chromatin structure as well as removing binding sites for proteins that contain bromodomains [17]. CHD3 and CHD4 contain subunits with ATP-helicase activity that remodels nucleosomes [18, 19]. This activity dynamically controls the accessibility of chromatin to DNA binding proteins and additional coregulators.

Other proteins within the core NuRD complex support and enhance these enzyme activities, as well as facilitating recruitment to chromatin. RBBP4 and RBBP7 (RbAp48/46) are ∼50 kDa WD40 domain proteins that interact with chromatin and have been shown to bind to histone H4 tails [20]. p66α and p66β (GATAD2A/B) contain GATA zinc finger domains and are involved in binding to unmodified histone tails [21]. MBD2 and MBD3 have been shown to bind to methylated cytosine-guanosine dinucleotides (CpGs) [22]. MBD2 has been found to localise at transcriptional start sites with methylated CpG islands, and this enrichment coincides with gene repression. MTA1 and the closely related MTA2 and MTA3 proteins enhance and direct the activity of HDAC1 to modify chromatin [1, 23]. MTA1 has been shown to directly interact with RBBP4, HDAC1 and CHD4 within the NuRD complex [24–27].

## MTA family proteins

MTA1 is an 80-kDa protein that has four conserved domains within the amino-terminal half of the protein. These are a bromo-adjacent homology (BAH) domain; an egl-27 and MTA1 homology domain 2 (ELM2); a Swi3, Ada2, NCoR and TFIIIB domain (SANT); and GATA-zinc finger domain (GATA) (Fig. 1). MTA1 and its homologues MTA2 and MTA3 share 63 and 72 % identity, respectively. The proteins are essentially identical within these four structured domains with the only notable difference being an extended loop within the MTA1-BAH domain. MTA1 is the largest protein in the family with 715 residues; MTA2 has 668 residues; and MTA3 has 594 residues. The carboxy-termini are significantly more divergent, which may explain why MTA1, MTA2 and MTA3 are found in mutually exclusive complexes and are implicated in different signalling pathways [14].

MTA1 can be differentially spliced, and the four conserved domains are truncated or missing in the resulting isoforms [28]. MTA1-short (MTA1s) is one of the best-characterised splice variants, being around half the size of full-length MTA1 and is truncated between the SANT and GATA domain. The original carboxy-terminus is replaced with a 33-residue extension containing the nuclear receptor box motif (LxxLL). The LxxLL motif interacts with the AF2 domain of ERα and results in the localisation of ERα to the cytoplasm in estrogen-positive breast cancer cells [29]. Another isoform known as ZG29p contains just the carboxy-terminus of MTA1 and is encoded by the last seven exons [30]. The resulting protein is missing all four conserved domains. A number of further isoforms have been characterised and confirm that the carboxy-terminus must contain at least one nuclear localisation signal for MTA1 to be retained within to the nucleus [28].

In addition to these four conserved domains, MTA1 contains two motifs that have the potential to promote SH3-binding and five SPXX motifs that are highly conserved across species [31–33]. Although these MTA1 motifs are yet to be fully characterised, considerable insight into the structure and function of these domains can be gained through sequence analysis. Comparison with related protein families can be used to better understand the assembly and function of the core NuRD corepressor complex.

## Structurally characterised domains of the MTA family

There are an increasing number of structures of MTA family domains in isolation and in complex with binding partners. The first structural information for the MTA proteins was a structure of the SANT domain from MTA3 that was deposited in the protein databank (PDB code 2CRG) but without any corresponding publication. More recently, Millard et al. determined the structure of the ELM2 and SANT domains from MTA1 bound to HDAC1, revealing the nature of the interface between the two proteins as well as highlighting a mechanism of regulation through inositol phosphate signalling [26]. Finally, a carboxy-terminal fragment of MTA1 has been crystallised bound to RBBP4 (RbAp48) [25]. Insights from the structural as well as functional studies of these domains are discussed below.

### The SANT domain and HDAC activation by inositol phosphates

The SANT domain is a relatively small domain of around 50 amino acids and is found in a number of proteins that are involved in transcription regulation [34]. The domain was originally identified in the nuclear receptor corepressor protein NCOR1 and in chromatin modifying proteins SWI3 and ADA2 [35]. The domain folds to form a three-helix bundle around a small hydrophobic core and is highly similar to the DNA-binding domain of Myb-related proteins.

**Fig. 2:**
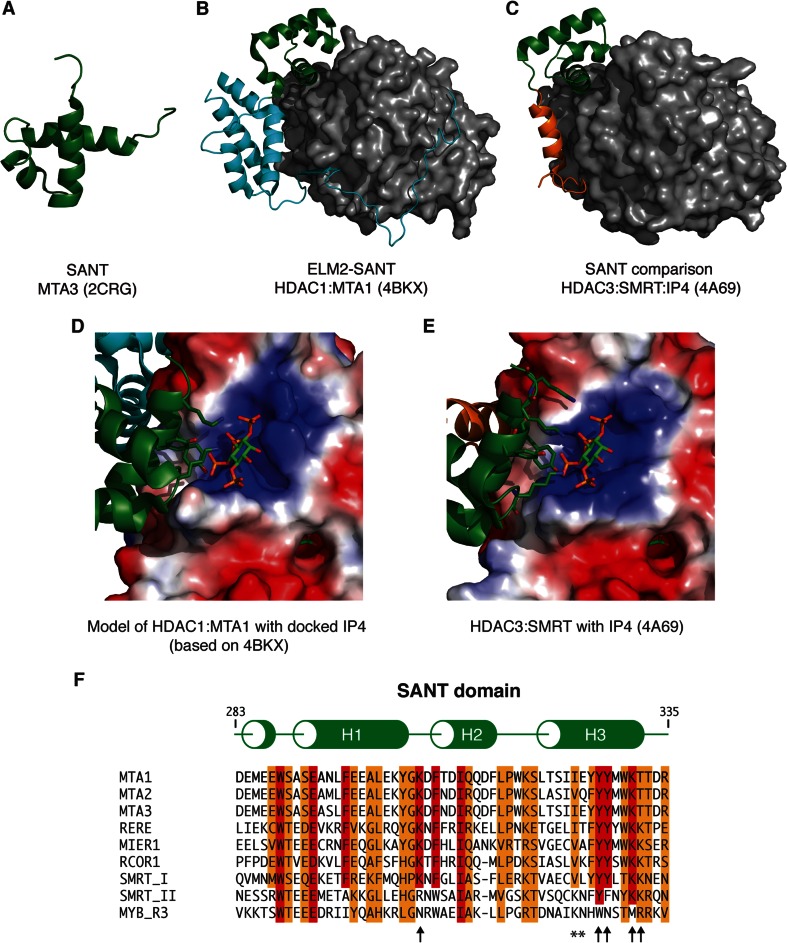
The SANT domain and its role in inositol phosphate signalling. **a** Solution structure of the SANT domain from MTA3 showing that the domain adopts a three-helix bundle. **b** Crystal structure of HDAC1 (*grey*) in complex with the ELM2 (*cyan*) and SANT (*green*) domains of MTA1. **c** Crystal structure of HDAC3 (*grey*) in complex with the SANT (*green*) domain of SMRT. **d**, **e** Electrostatic surface representations of HDAC1 and HDAC3 with their respective corepressor proteins MTA1 and SMRT. IP4 is modelled into the basic binding pocket at the interface between HDAC1 and MTA1 in the same orientation as observed in the HDAC3:SMRT crystal structure. **f** Sequence alignment of SANT domains from other corepressor proteins. The MYB-DNA binding domain is included for comparison. *Arrows* indicate the IP4 interacting residues in the HDAC3:SMRT structure, and *stars* indicate the residues in MYB-R3 domain that mediate interaction with DNA (colour figure online)

The NMR structure of the MTA3-SANT domain confirmed the predicted helix-turn-helix arrangement (Fig. 2). Inspection of the structure suggests that the SANT domain is functionally divergent from the Myb DNA-binding domain [36]. Important basic DNA recognition residues within helix 3 (or the recognition helix) are not conserved, suggesting that the SANT domain would be unable to make sequence-specific contacts to DNA. In addition, the SANT domain contains three bulky hydrophobic residues at the carboxy-terminus of helix 3 that would sterically hinder DNA binding. This suggests that, despite the conserved fold, these domains have evolved to perform different functions.

More recently, the structure of a longer fragment of MTA1 that includes both the ELM2 and SANT domains bound to HDAC1 has been solved by X-ray crystallography. This structure shows that helix 3 of the SANT domain forms a basic pocket at the interface between HDAC1 and MTA1, near the active site of the enzyme [26]. This pocket has implications for enzyme activation of class I HDACs (see below).

Sequence alignments show that the SANT domain of MTA1 is highly related to the first of two SANT domains from the nuclear receptor corepressor protein SMRT (also known as NCOR2). The SMRT-SANT is a critical partner of HDAC3 and is essential for HDAC3 activity [37]. The structure of HDAC3 bound to SMRT-SANT has been solved and shows that a inositol-(1,4,5,6)-tetrakisphosphate (IP4) molecule can bind into a basic pocket at the interface between the two proteins [38]. Importantly, IP4 has a key role in activating the enzyme, since high salt treatment causes HDAC3 to become inactive and adding back exogenous IP4 leads to enhanced HDAC activity. The concentration of IP4 required for activation of HDAC3 is comparable to the measured levels of inositol phosphates in the cell [39].

The residues coordinating IP4 binding in HDAC3 and SMRT are conserved in HDAC1 and MTA1-SANT, and a similar basic pocket is present at the interface between the two proteins. This pocket is formed through the contribution of a lysine and two arginine residues from HDAC1, and two lysine and two tyrosine residues from MTA1. The similarity in size and charge of the pocket suggested that an inositol phosphate molecule could be accommodated. However, in the crystal structure, an inositol phosphate molecule is not present. Native mass spectroscopy showed that the inositol phosphate is lost during purification and is replaced during crystallisation by ordered sulphate molecules (2 M ammonium sulphate was used as a precipitant). As with HDAC3:SMRT, the addition of IP4 to HDAC1:MTA1 enhances HDAC activity, and mutation of the IP4 coordinating residues inhibits activation [26].

Interestingly, the inositol phosphate interacting residues are not only conserved in SMRT-SANT and MTA-SANT domains but also in related corepressor proteins such as RCOR1-3, MIER1-3 and RERE. These SANT domain-containing proteins are recruited to distinct corepressor complexes that recruit HDAC1, and all could potentially form an inositol-binding pocket. Since HDAC1 and HDAC2 are highly homologous, the binding of inositol phosphates will be a common activating mechanism for HDAC1, 2 and 3 (*i.e.* all class I HDAC corepressor complexes).

### The ELM2 domain and dimerisation of the NuRD complex

The ELM2 domain within corepressor proteins such as MTA1-3, RCOR1-3, MIER1-3 and RERE has been shown to be required for the recruitment of HDAC1 and HDAC2 to the respective corepressor complexes. The ELM2 domain is positioned immediately amino-terminal to the SANT domain. Structure prediction of the MTA1-ELM2 domain suggests that the domain is largely intrinsically disordered (*i.e.* lacking an intrinsically fixed structure), although there are predicted helical regions at the carboxy-terminus. Circular dichroism experiments show that the ELM2 domain contains essentially no secondary structure when expressed in isolation [26]. However, when coexpressed with HDAC1, the ELM2-SANT domain forms a stable complex with the HDAC enzyme [26].

The crystal structure of the complex between HDAC1 and the ELM2-SANT domain from MTA1 shows that the ELM2 domain is divided into two structural regions [26]. The amino-terminal part of the ELM2 domain adopts an extended conformation that wraps around the HDAC making multiple interactions. Close to the amino-terminus, there is a conserved sequence (consensus: EIRVGxxYQAxI; residues 166–177) that binds in a conserved groove on the HDAC. This is positioned close to the active site of the HDAC enzyme [26].

**Fig. 3:**
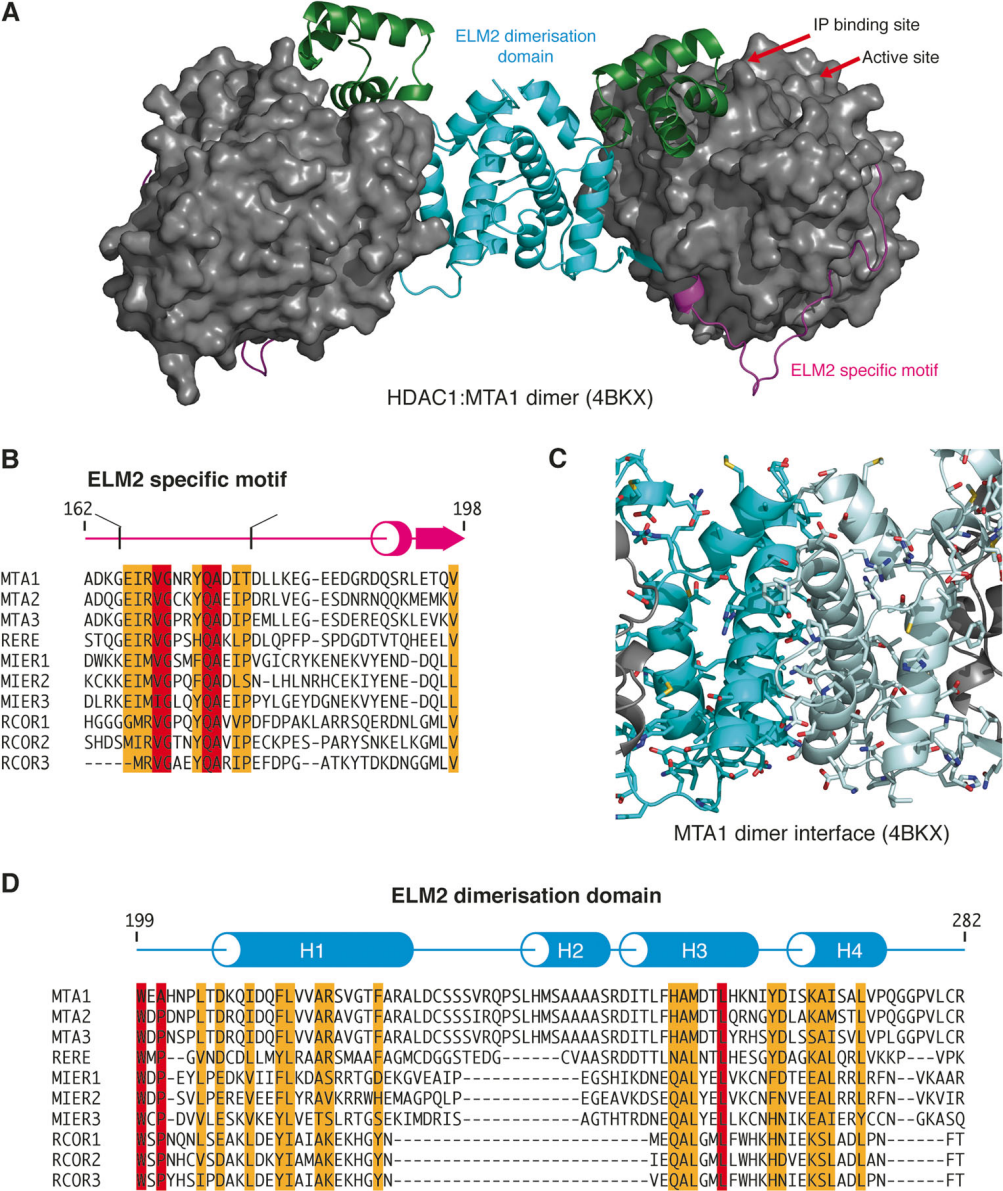
The dimeric ELM2 domain of MTA1. **a** Structure of dimeric MTA1 bound to two copies of HDAC1 (*grey*). MTA1 is coloured by ELM2-specific motif (*magenta*), ELM2 dimerisation domain (*cyan*) and SANT domain (*green*). **b** Alignment of the amino-terminus of ten ELM2 domains highlighting the conservation of the ELM2-specific motif. **c** Structure of the MTA1 dimer interface with side chains shown as sticks. **d** Sequence alignment of ELM2 dimerisation domain with other corepressor proteins. The secondary structure of MTA1 is shown above with *cylinders* indicating α-helices and the *arrow* indicates β-strands (colour figure online)

The carboxy-terminal region of the ELM2 domain forms a four-helix bundle with a small hydrophobic core and enlarges the interacting surface with HDAC1 as it completes a path around the ‘back’ of the enzyme. The four-helix bundle forms a homodimer and is therefore able to associate with a second HDAC1:MTA1 complex (Fig. 3). The homodimerisation interface is extensive with 14 non-polar side chains being contributed from each ELM2 domain. The highly complementary nature of this interface suggests that it is physiologically relevant. This means that the NuRD complex contains two copies of both HDAC1 and MTA1 proteins, and given that HDAC1 and HDAC2 are highly similar (83 % identical), it is likely that both HDACs can coexist in a single NuRD complex.

The dimerisation of HDAC1:MTA1 (through the ELM2 domain) positions the active site of the two HDACs on approximately the same face of the dimer. This orientation situates the two active sites approximately 90 Å apart and could allow the complex to simultaneously target more than one nucleosome.

### The carboxy-terminus and interaction with RBBP4

RBBP4 and RBBP7 are WD40-repeat proteins that share 92 % identity and are integral components of the NuRD complex [2]. These proteins act as histone chaperones and associate with additional chromatin modifying complexes including SIN3A, PRC2 and NURF [40–42]. RBBP4 and RBBP7 have a seven-bladed β-propeller architecture and have at least two distinct binding sites for partner proteins: the first on the top of the protein (structurally characterised as the binding site for histone H3 and FOG1) and the second on the side of the protein, involving α-helices at both the amino- and carboxy-termini and an extended loop inserted into blade 6 (the binding site for histone H4 and Su(z)12) [20, 43, 44].

**Fig. 4:**
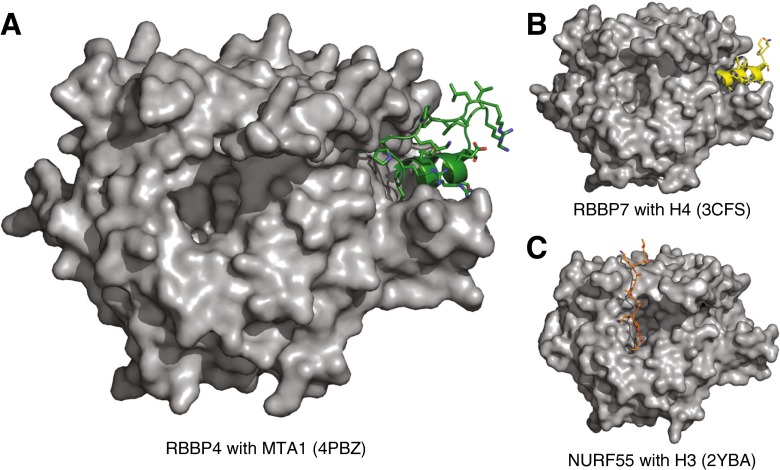
Histone tail interactions with WD40 domain containing proteins. **a** MTA1 (residues 670–691, shown in *green*) bound in a groove on the side of RBBP4. This groove is formed by the amino- and carboxy-termini and a loop from blade 6. **b** Histone H4 (*yellow*) bound to RBBP7 in the same groove as MTA1. **c** Histone H3 (*magenta*) has a distinct binding site on WD40 domain as shown in the structure of the *Drosophila* homologue NURF55. Histone H3 is likely to have a similar binding site on top of the WD40 domain of RBBP4/7. All WD40 domains are shown in the same orientation (colour figure online)

Several studies have used GST pull-down assays to map the interaction between MTA1/2 and RBBP4/7. These have suggested that there may be three non-overlapping regions in the carboxy-terminus of MTA1 that mediate this interaction. One of these includes the GATA-type zinc finger domain [45, 46]. More recently, a short motif at the carboxy-terminus of MTA1 (residues 656–686) was structurally characterised as being able to bind to RBBP4 [25] (Fig. 4). This fragment is helical on binding to RBBP4 and binds in an acidic groove on the side of the WD40 domain. This groove was previously shown to bind to histone H4 [20]. Indeed, both fragments of MTA1 and histone H4 include the motif KRAARR and both form analogous contacts to RBBP4. Competition assays showed that MTA1 and histone H4 compete for the same binding site on RBBP4, suggesting that MTA1 modulates RBBP4 interaction with histones.

The second binding site was observed in the structure of the *Drosophila* homologue Nurf55 bound to a histone H3 tail peptide. In this structure, a peptide corresponding to histone H3 binds at the top of the propeller [43]. There is no overlap between the H3 and H4 binding sites, suggesting that MTA1 only competes for binding with histone H4. RBBP4 and RBBP7 are found in many corepressor complexes such as SIN3A, PRC2 and NURF, and the two distinct binding sites would increase their ability to recruit alternate binding partners in different settings.

## Structural insight into other domains of the MTA family

MTA contains additional domains and motifs that remain to be structurally and functionally characterised. These domains have been identified through sequence alignment with other characterised proteins. Through sequence comparison and modelling, we can speculate on how these domains will look and how they will function.

### The BAH domain

Hydrophobic cluster sequence analysis identified a single copy of the BAH domain in MTA1 that spans residues 1–164 [47]. The BAH domain was first described in the protein Polybromo, a subunit of a 2-MDa chromatin remodelling complex. Polybromo contains two BAH domains carboxy-terminal to four bromodomains [48]. The BAH domain has also been identified in a number of proteins involved in transcriptional regulation including the yeast RSC chromatin remodelling complex proteins (RSC1 and RSC2) and the transcription factor ASH1. BAH domains are also found in ORC1, SIR3 and DNMT1 proteins. Sequence alignments show that there are approximately 130 conserved amino acids within BAH domains although some contain large insertions of variable length.

**Fig. 5:**
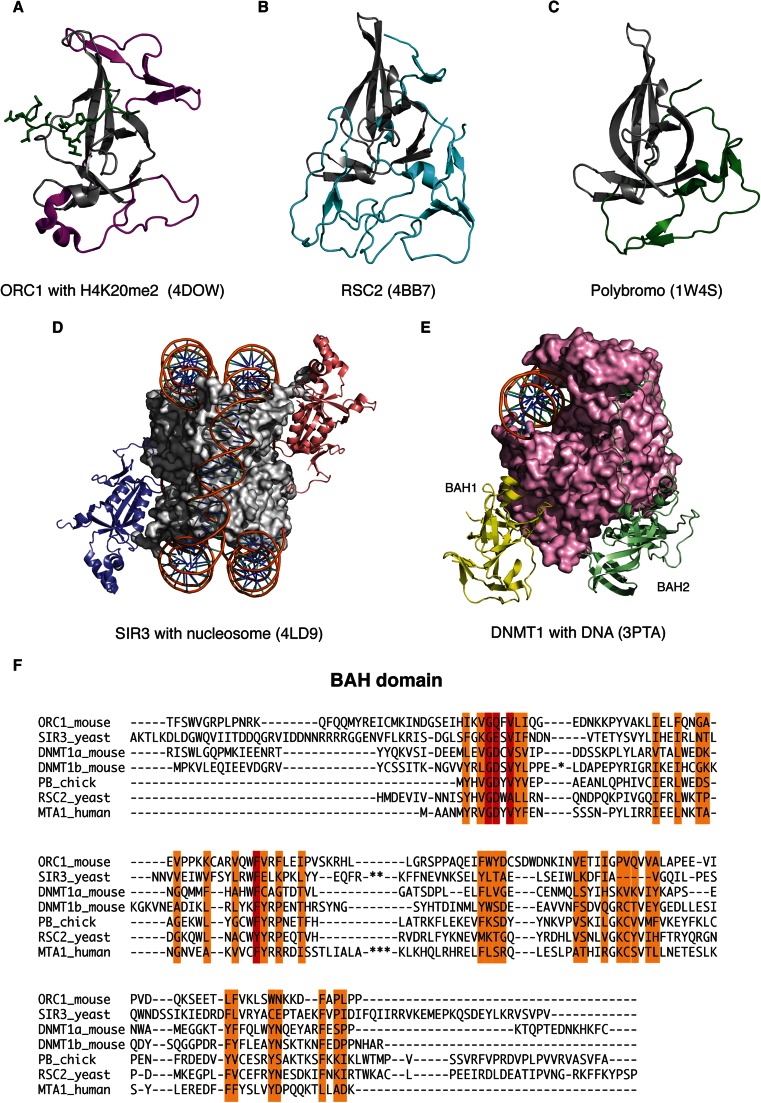
The structure of the BAH domain. **a** The BAH domain from ORC1 (*magenta*) with bound methylated H4 peptide (*green*). **b** The BAH domain from RSC2 (*cyan*). **c** The BAH domain Polybromo (*green*). The canonical core BAH domain is coloured *grey* in each case. **d** Two copies of SIR3-BAH (*purple* and *salmon*) in complex with a nucleosome. DNA is shown as a cartoon, and the four histones are shown as surface (*grey*). **e** DNMT1 bound to DNA. The BAH1 and BAH2 domains are highlighted (*yellow* and *light green*), and the rest of the protein is shown as surface (*pink*). **f** Alignment of the MTA1-BAH with the sequences of BAH domains for which their structure is known. Forty residues in DNMT1b (*), 15 residues in SIR3 (**) and 38 residues in MTA1 are not shown (***) (colour figure online)

Several structures of BAH domains have been solved and show that the core of the domain is largely β-sheet in character (Fig. 5). Beyond this core, each protein has a divergent fold including 3_10_ helices, β-strands and ordered loops. This diversity may be a requirement of each protein to perform different functions. Structure prediction suggests that MTA1 will fold to form a β-barrel core with two long insertions.

The BAH domain of MTA1 is most closely related to the two BAH domains of Polybromo. The proximal BAH domain of Polybromo has a six-stranded β-barrel core, the centre of which is completely filled with hydrophobic residues [49]. A key feature of the domain is the long carboxy-terminal extension that effectively ‘wraps’ around the outside of the protein to stabilise two extended loops and the rest of the protein. Sequence comparison of the Polybromo-BAH domain with MTA1 suggests that the MTA1-BAH carboxy-terminal extension is shorter and is immediately followed by the ELM2 domain.

The BAH domains in RSC2, ORC1 and SIR3 have been shown to mediate interactions with chromatin. If this were also the case for the BAH domain from MTA1, this would provide an attractive explanation as to how chromatin is recruited to the corepressor complex allowing the HDAC to target acetylated lysines in the histone tails.

The BAH domain from the yeast RSC2 protein has been shown to bind histone H3 [50]. However, sequence comparison shows that the two tryptophan residues in the RSC2-BAH involved in binding the unmodified histone H3 peptide are not conserved in the MTA1-BAH domain suggesting that MTA1 cannot bind histone H3 in the same way as RSC2.

The yeast SIR3 protein not only interacts with histone tails but the BAH domain also makes extensive contacts with the nucleosome [51–54]. The crystal structure of the complex shows that acetylated amino-terminus of SIR3-BAH domain is critical for interaction with histones H4 and H2B [53, 54]. The amino acids in SIR3 that mediate interactions with the nucleosome show very limited conservation in the MTA1-BAH domain, making it difficult to predict that MTA1 would bind nucleosomes in an analogous fashion.

The ORC1-BAH domain has also been shown to bind to histone H4. In this case, binding only occurs when the histone tail is dimethylated at lysine 20 (H4K20me2) [55]. The dimethylated side chain is bound within an aromatic cage on the surface of the ORC1-BAH domain. Again, sequence comparisons with the MTA1-BAH domain show that tyrosine and tryptophan residues important for binding H4K20me2 are not conserved.

These structures support the BAH domain as a histone-binding domain but the diversity within the family only allows a tentative suggestion that the BAH domain of MTA1 could bind to histone tails. Indeed, the two BAH domains from the chromatin-associated DNMT1 methyltransferase have not been shown to mediate direct interactions with chromatin [56]. Instead, they appear to contribute indirectly to substrate recognition by DNMT1.

Whilst it remains to be seen whether the MTA1-BAH domain directly recruits chromatin, the location of the domain immediately adjacent to the ELM2 domain means that it will be positioned very close to the active site of the HDAC enzyme [26]. Therefore, it seems inevitable that it will play some role in substrate interaction.

### GATA-type zinc finger domain

**Fig. 6:**
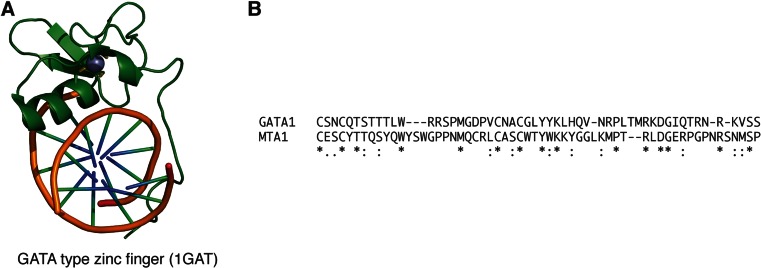
The GATA1 zinc finger bound to DNA. **a** The fold of the domain (*green*) is stabilised by the central zinc atom (*grey sphere*). **b** Sequence alignment showing the residues conserved between GATA1 and the predicted zinc finger domain of MTA1 (residues 393–450) (colour figure online)

MTA1 contains a GATA-type zinc finger domain positioned carboxy-terminal to the SANT domain. GATA-type zinc fingers are found in many eukaryotic transcription factors where they contribute to sequence-specific DNA recognition [57]. The zinc finger domain is composed of around 30 amino acids with four conserved cysteines coordinating a structural zinc ion [58] (Fig. 6). The domain interacts directly in the major groove of the DNA but also makes many interactions with the phosphate backbone of the DNA. The carboxy-terminus interacts in the adjacent minor groove of the DNA [58].

Sequence comparison shows that the GATA-type zinc finger in MTA1 is rather different from GATA1. It is likely that it retains DNA-binding activity, but given the lack of conservation of the relevant residues, it will probably either recognise a different sequence from GATA1 or, perhaps more likely, may bind to DNA without sequence specificity.

### Additional domains and intrinsically disordered regions

MTA1 contains additional motifs that remain to be structurally and functionally characterised [31]. The xPPxP motif appears twice in MTA1 and could potentially be recognised by SH3 domains. The SH3 binding motif (xPPxP) is recognised by a 60 amino acid domain that is widely found in cytoskeletal elements and signalling proteins [59]. The proline-rich motif typically adopts a left-handed polyproline helix conformation and is recognised by the SH3 domain with characteristic β-barrel fold [60].

Towards the carboxy-terminus of MTA1, there are five SPXX motifs and these have been proposed to be sites of MTA1 phosphorylation [61]. The phosphorylation pattern on MTA1 was shown to dynamically change at each of these sites during the first 24 h of differentiation. One of the short forms of MTA1 (MTA1s) has been characterised as being phosphorylated by casein kinase [62]. The phosphorylation of MTA1 at this site was shown to be important for repressing estrogen-induced ER transactivation.

Further MTA1 motifs include a methylation site, which has shown to be involved in recruiting CHD4 and two nuclear localisation motifs [27, 63].

## Conclusions

MTA1 is a key scaffold protein in the NuRD corepressor complex and plays a vital role in both assembling the complex and activating the HDAC enzymes. MTA1 directly contacts many of the core NuRD components. A number of these interactions occur within the structurally conserved domains of MTA1, but others are directed through intrinsically disordered regions that become ordered on binding and are key to forming a stable NuRD complex.

Three domains of MTA1 have now been characterised by NMR and X-ray crystallography. The ELM2 and SANT domains have been shown to recruit HDAC1 and/or HDAC2 through an extensive interface that excludes 1,278 Å^2^ from solvent exposure [26]. The extensive nature of this interface begins to address why the NuRD complex is so stable, as the components have previously been shown to remain intact on isolation from cells and show resistance to high salt treatment [3]. The stability of a core NuRD complex appears to be a common feature in other corepressor complexes such as the NCoR, SIN3 and PRC2 complexes [40, 41, 64].

The other structurally characterised motif (KRAARR) of MTA1 lies towards the carboxy-terminus. This motif has been shown to interact with RBBP4 and modulates binding to histone H4 [25]. This competition highlights the role of MTA1 as an essential scaffold component of the NuRD complex that mediates many key interactions.

In addition to its role as a scaffold protein, MTA1 also plays an important role in activating HDACs through forming one half of the binding site for inositol phosphates that have been shown to serve as activator molecules for class I HDACs.

Intriguingly, a many of the core NuRD complex proteins have the potential to interact with DNA and chromatin. The RBBP, p66, MTA and MBD proteins all have domains that are likely to interact with either histone tails, nucleosome or DNA. This permits the NuRD complex to use a large repertoire of modules to target nucleosome remodelling and deacetylase activity to chromatin. A full understanding of the activity and mechanism of the NuRD complex will require more challenging structural studies of the intact complex and how it interacts with its chromatin substrate. Such studies are likely also to provide further insights into strategies to target the complex therapeutically.
